# Eccentricity-dependent temporal contrast tuning in human visual cortex measured with fMRI

**DOI:** 10.1016/j.neuroimage.2018.09.049

**Published:** 2019-01-01

**Authors:** Marc M. Himmelberg, Alex R. Wade

**Affiliations:** a Department of Psychology, The University of York, Heslington, York, YO10 5DD, United Kingdom; b York NeuroImaging Centre, The Biocentre, York Science Park, Heslington, York, YO10 5NY, United Kingdom

**Keywords:** Visual cortex, Temporal contrast sensitivity, Magnocellular pathway, Eccentricity, fMRI, Population receptive fields

## Abstract

Cells in the peripheral retina tend to have higher contrast sensitivity and respond at higher flicker frequencies than those closer to the fovea. Although this predicts increased behavioural temporal contrast sensitivity in the peripheral visual field, this effect is rarely observed in psychophysical experiments. It is unknown how temporal contrast sensitivity is represented across eccentricity within cortical visual field maps and whether such sensitivities reflect the response properties of retinal cells or psychophysical sensitivities. Here, we used functional magnetic resonance imaging (fMRI) to measure contrast sensitivity profiles at four temporal frequencies in five retinotopically-defined visual areas. We also measured population receptive field (pRF) parameters (polar angle, eccentricity, and size) in the same areas. Overall contrast sensitivity, independent of pRF parameters, peaked at 10 Hz in all visual areas. In V1, V2, V3, and V3a, peripherally-tuned voxels had higher contrast sensitivity at a high temporal frequency (20 Hz), while hV4 more closely reflected behavioural sensitivity profiles. We conclude that our data reflect a cortical representation of the increased peripheral temporal contrast sensitivity that is already present in the retina and that this bias must be compensated later in the cortical visual pathway.

## Highlights

• Contrast sensitivity to 20Hz flicker is increased in peripheral voxels, when compared to foveal voxels, within V1, V2, V3, and V3a.
• In hV4, contrast sensitivity at 20Hz is unchanged across eccentricity, similar to psychophysical sensitivities.
• Our data suggest that the temporal response properties of peripheral retinal cells are represented in the cortical periphery of early visual areas.
• Temporal filtering may occur in higher-order ventral visual areas, consistent with an eccentricity-dependent invariance to lower level visual properties such as temporal contrast.

## 1 Introduction

There is a mismatch between electrophysiological retinal measurements and psychophysical measurements of temporal contrast sensitivity across the visual field. Eccentricity-dependent differences in retinal temporal sensitivity originate in the cone photoreceptors – peripheral cones respond faster and are more sensitive to flicker when compared to those in the fovea (Sinha et al., 2017). These signals are filtered through the retinal ganglion cells (RGCs), where there is an increase in the proportion of parasol to midget RGCs with increasing retinal eccentricity (Connolly and van Essen, 1984; Dacey, 1993, 1994; Dacey and Petersen, 1992; De Monasterio and Gouras, 1975). Temporal frequency sensitivity is thought to be related to the relative activity of parasol to midget RGC populations which form the magnocellular and parvocellular pathway, respectively (Hammett et al., 2000; Harris, 1980). On average, RGCs in the periphery have larger receptive fields and cells with such receptive fields have increased contrast sensitivity (Dacey and Petersen, 1992; Enroth-Cugell and Shapley, 1973). Overall then, the peripheral retina has relatively more parasol cells, those cells integrate from larger portions of the retina, and they are fed by cones with brisker response kinetics (Dacey and Petersen, 1992; Enroth-Cugell and Shapley, 1973; Sinha et al., 2017). From such physiological differences we might expect subjects to be more sensitive to low contrast flickering stimuli in more peripheral regions of the visual field.

These predictions are not generally confirmed by psychophysical measurements of temporal contrast sensitivity across space. Previous research has found that psychophysical temporal contrast thresholds are approximately independent of visual field eccentricity (Koenderink et al., 1978; Virsu et al., 1982; Wright and Johnston, 1983). Although such thresholds (which by definition, occur at relatively low contrast) are independent of eccentricity, very low spatial frequencies might be an exception: previous papers report an increase in critical flicker frequency with increasing eccentricity (Hartmann et al., 1979; Rovamo and Raninen, 1984). How these eccentricity-dependent sensitivities to temporal contrast are represented in the visual cortex is currently unknown.

The early visual cortex is organised retinotopically; visual space is mapped topographically, with foveal receptive fields mapped towards the occipital pole and more peripheral receptive fields mapped in increasingly anterior areas of the cortex (Engel et al., 1997). Perhaps then, investigating sensitivity to temporal contrast across cortical space can help to explain the discrepancy between measurements of retinal and psychophysical temporal contrast sensitivity. Previous research has found centrally located sustained and peripherally located transient temporal channels in primary visual cortex, and these channels are thought to reflect responses from different classes of cells (Horiguchi et al., 2009). One might ask whether the relative weighting of response properties of peripheral retinal cells to temporal frequency and contrast is maintained in V1 and other early visual areas. One might also ask at what point in the cortical pathway is temporal contrast sensitivity filtered to reflect psychophysical sensitivity across space, rather than retinal sensitivity. One might expect such filtering to occur in higher-order visual areas that are typically specialized for complex feature identification computations, and are less reliant on temporal frequency and contrast information.

How do measurements of cortical temporal contrast sensitivity differ across visual space, and how do such cortical sensitivities relate to behaviour? To answer this, we used fMRI to measure voxel contrast response functions (CRFs) at a range of temporal frequencies and plotted responses as a function of pRF eccentricity in different visual areas. Additionally, we obtained psychophysical temporal contrast threshold measurements in central and near-peripheral regions of visual space. Previous research has found that the optimal contrast sensitivity of the primate visual system is approximately 8 Hz, thus we predicted that we would observe a similar peak contrast sensitivity, independent of eccentricity, in our psychophysical and fMRI data (Hawken et al., 1996; Kastner et al., 2004; Singh et al., 2000; Venkataraman et al., 2017). Next, due to retinal biases, we predicted that in early visual areas contrast sensitivity would be greater at a high temporal frequency in pRFs representing more peripheral locations of the visual field. Conversely, if cortical sensitivities are to shift to be more reflective of behaviour at some point in the visual cortex, it is predicted that such areas will show no difference in temporal contrast sensitivity across pRF eccentricity.

## 2 Materials and methods

### 2.1 Participants

Nineteen participants (mean ± SD age, 27.89 ± 5.72; 9 males) were recruited from the University of York. All participants had normal or corrected to normal vision. Each participant completed a 1-h psychophysics session and two 1-h fMRI sessions. In the first fMRI session, two high-resolution structural scans and six pRF functional runs were obtained. In the second fMRI session, 10 temporal contrast sensitivity (TCS) functional runs were obtained. All participants provided informed consent before participating in the study. Experiments were conducted in accordance with the Declaration of Helsinki and the study was approved by the ethics committees at the York NeuroImaging Centre and the University of York Department of Psychology.

### 2.2 Behavioural psychophysics

#### 2.2.1 Experimental design

To investigate psychophysical temporal contrast sensitivity, we measured contrast detection thresholds for four temporal frequency conditions (1, 5, 10, and 20 Hz) at two eccentricities (2° and 10°). 75% correct detection thresholds were obtained using a ‘2 Alternative Forced Choice’ (2AFC) method using four randomly interleaved Bayesian staircases in separate eccentricity blocks (Kontsevich and Tyler, 1999). A single block of 200 trials (50 of each temporal frequency condition) was presented at either 2° or 10° from central fixation on the temporal visual field meridian. Participants were instructed to maintain fixation on a central cross and to respond, via keyboard press, whether the stimulus grating appeared on the left or right of fixation. Participants were informed via a toned ‘beep’ if their response was correct or incorrect. These responses were recorded using Psykinematix software (KyberVision, Montreal, Canada, psykinematix.com). After each response, a separate toned ‘beep’ was presented in conjunction with the fixation crossed briefly changing to ‘o’ then back to ‘x’ to signify the onset succeeding trial, which then began 500 ms later. The first 10 trials were practice and not included in the analysis. The temporal frequency of the stimulus was randomized within each block. Participants completed each eccentricity condition block four times and responses were fit with Weibull functions of stimulus contrast. This resulted in four 75% contrast detection thresholds for each temporal frequency and eccentricity combination. For each condition, the average of these 4 thresholds was the final threshold.

#### 2.2.2 Stimuli

**Fig. 1:**
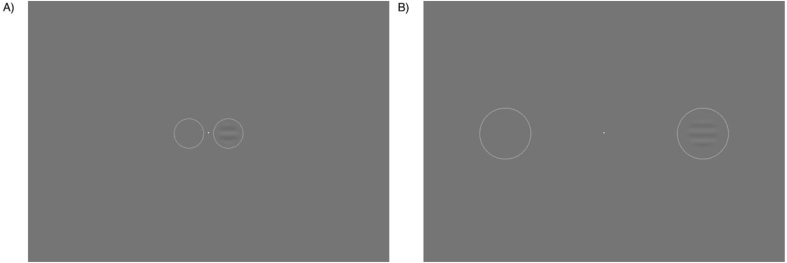
2AFC stimulus at two eccentricity conditions. In A) a flickering stimulus grating appears in the right circle at 2° eccentricity, while in B) the flickering stimulus grating appears in the right circle at 10° eccentricity. Participants must select which circle the grating appears in.

Psychophysical stimuli (see Fig. 1) were designed using Psykinematix software and were presented on a NEC MultiSync 200 CRT monitor running at 120 Hz. Gamma correction was performed using a ‘Spyder5Pro’ (Datacolor, NJ, USA) display calibrator. Stimuli were circularly windowed sine wave gratings outlined with thin white circles to eliminate spatial uncertainty (Pelli, 1985). Grating spatial frequency was set to 1 cycle per degree (cpd) and were presented for 500 ms. At 2° eccentricity, the grating had a 0.5° radius. Using M-scaling to account for cortical magnification, at 10° eccentricity the stimulus had a 1.021° radius (Rovamo and Virsu, 1979).

### 2.3 Functional neuroimaging

#### 2.3.1 fMRI stimulus display

Stimuli were presented in the scanner using an PROpixx DLP LED projector (VPixx Technologies Inc., Saint-Bruno-de-Montarvile, QC, Canada) with a long throw lens that projected the image through the waveguide behind the scanner bore and onto an acrylic screen. The image presented had a resolution of 1920 × 1080 and a refresh rate of 120 Hz. Participants viewed this screen at a viewing distance of 57 cm using a mirror within the scanner. Gamma correction was performed using a customized MR-safe ‘Spyder4’ (Datacolor, NJ, USA) display calibrator.

#### 2.3.2 fMRI data acquisition

Scans were completed on a GE Healthcare 3 T Sigma HDx Excite scanner (GE Healthcare, Milwaukee, WI). Structural scans were obtained using an 8-channel head coil (MRI Devices Corporation, Waukesha, WI) to minimize magnetic field inhomogeneity. Functional scans were obtained with a 16-channel posterior head coil (Nova Medical, Wilmington, MA) to increase signal-to-noise in the occipital lobe.

#### 2.3.3 Pre-processing of structural and functional scans

Two high-resolution, T1-weighted full-brain anatomical structural scans were acquired for each participant (TR, 7.8 ms; TE, 3.0 ms; TI, 450 ms; voxel size, 1.3 × 1.3 × 1 mm^3^; flip angle, 20°; matrix size, 176 × 256 x 257). To improve grey-white matter contrast, the two T1 scans were aligned and then averaged together using FSL tool FLIRT (Jenkinson et al., 2012). This averaged T1 was automatically segmented using a combination of FreeSurfer (http://surfer.nmr.mgh.harvard.edu/) and FSL, and manual corrections were made to the segmentation using ITK-SNAP (http://www.itksnap.org/pmwiki/pmwiki.php) (Teo et al., 1997). At the beginning of each functional session, one 16-channel coil T1-weighted structural scan with the same spatial prescription as the functional scans was acquired to aid in the alignment of functional data to the T1-weighted anatomical structural scan.

Functional data were pre-processed and analysed using MATLAB 2016a (Mathworks, MA) and VISTA software (https://vistalab.stanford.edu/software/) (Vista Lab, Stanford University). Between and within scans motion correction was performed to compensate for any motion artefacts that occurred during the scan session. Any scans with >3 mm movement were removed from the analysis. This resulted in the removal of one pRF run for two participants and one temporal contrast sensitivity scan for three participants. Functional runs were averaged across all scans. Next, we used mrVista tool rxAlign to co-register the 16-chanel coil T1-weighted structural scan to the 8-channel coil T1-weighted full-brain anatomical scan. First, we applied a manual alignment by using landmark points to bring the two volumes into approximate register. Next, we used a robust EM-based registration algorithm as described by Nestares and Heeger (2000) to fine tune the alignment. The final alignment was checked by eye to ensure that the automatic registration procedure optimised the fit. This alignment was used as a reference to align our functional data to our full-brain anatomical scan. These functional data were then interpolated to the anatomical segmentation.

#### 2.3.4 Population receptive field mapping scans

**Fig. 2:**
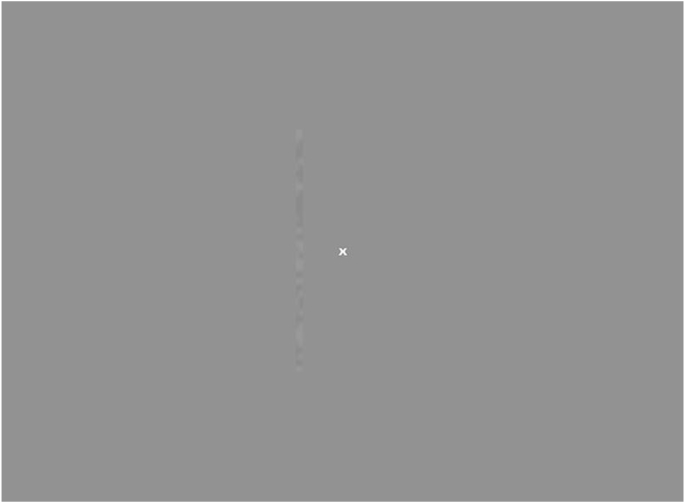
Example of the stimulus used to obtain pRF parameter estimates. The carrier is filled with pink noise that updates at 2 Hz as it drifts across the screen in 8 directions within a circular aperture with a 10° radius.

pRF scan sessions consisted of six 6.5-min pRF stimulus presentation runs collected using a standard EPI sequence (TR, 3000 ms; TE, 30 ms; voxel size, 2 × 2 × 2.5 mm^3^, flip angle 20°; matrix size, 96 × 96 x 39). Here, a drifting pRF bar stimulus was used to obtain retinotopic maps and estimates of pRF parameters (Dumoulin and Wandell, 2008). A single bar (width 0.5**°**) was swept in one of eight directions within a circular aperture (10° radius) with each sweep lasting 48 s. Using the conversion of visual angle to retinal eccentricity, 10° radius corresponds to mapping 2.83 mm radius retinal space (Drasdo and Fowler, 1974). To stimulate a broad population of neurons, the pRF carrier consisted of pink noise at 5% contrast, where the noise pattern changed at 2 Hz (see Fig. 2). A 12 s (4 TR) dummy run was included at the beginning of each functional run to allow for the scanner magnetization to reach a steady state. To maintain fixation throughout the scan, participants completed an attentional task where they responded, via button press, when the orientation of the fixation cross changed. This task was set up so that on average, every 2 s there was a 30% chance of a change in the orientation of the fixation cross.

**Fig. 3:**
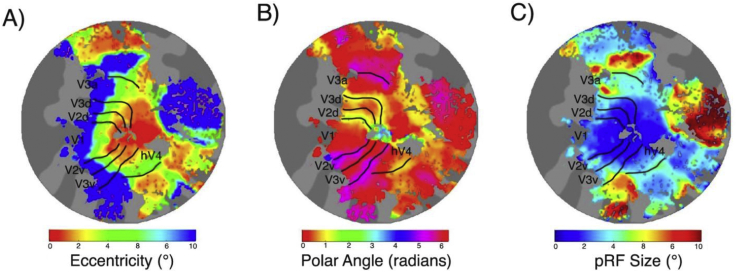
Exemplar left hemisphere retinotopic maps with ROI border overlays presented on flattened cortical representations for one subject. In A) we present eccentricity maps in which pRF eccentricity increases with distance from the fovea. In B) we present polar angle maps with border overlays based on polar angle reversals. In C) we present pRF size maps, that show an increase in pRF size within and between ROIs.

Using mrVista, pRF positions (i.e. eccentricity and polar angle parameters) and sizes were estimated for each voxel using the standard pRF model (Dumoulin and Wandell, 2008). In Fig. 3 we present exemplar eccentricity, polar angle, and pRF size maps from one participant. Following the nomenclature of Wandell et al. (2007) we delineated five bilateral regions of interest (ROIs); V1, V2, V3, V3a, and hV4, by hand on cortical flat maps based on polar angle reversals for each participant (see Fig. 3B).

### 2.4 Temporal contrast sensitivity (TCS) functional scans

#### 2.4.1 Stimulus

**Fig. 4:**
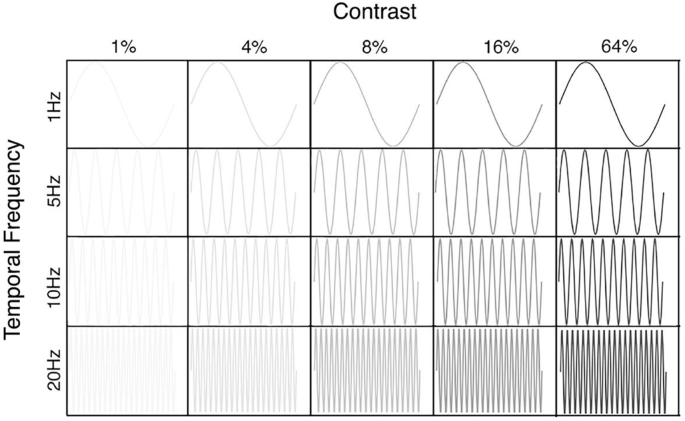
Visual representation of temporal contrast stimulus conditions. The sine wave grating sweeps through 20 temporal contrast conditions, with each condition being presented once per run for 3 s.

To investigate voxel temporal contrast sensitivity, we presented participants with a vertically oriented contrast reversing sine grating within a circular aperture (10° radius). The stimulus was generated and presented using MATLAB 2016a and Psychtoolbox v.3.0.13 (Brainard, 1997). We modulated both the contrast and temporal frequency of the grating. Within each functional run the sine wave grating was presented at 20 condition combinations of Michelson contrast (1, 4, 8, 16, and 64%) and temporal frequency flicker (1, 5, 10, and 20 Hz) (Michelson, 1927). The spatial frequency of the grating was held at 1 cpd. Each stimulus condition was presented once per run and lasted 3 s. A baseline condition of mean luminance was presented for 3 s during each run. Here, a single contrast reversal was defined as one complete on-off cycle off the stimulus. A visual representation of the experimental design is illustrated in Fig. 4.

#### 2.4.2 Data acquisition and analysis

TCS functional scan sessions consisted of ten 3.5-min stimulus presentation runs collected using an almost identical EPI sequence to that used for the pRF mapping (TR, 3000 ms; TE, 30 ms; voxel size, 2 × 2 × 2.5 mm^3^, flip angle 20°; matrix size, 96 × 96 x 39). The stimulus was presented using an event related design in which condition ordering was randomized within each run. A randomized interstimulus interval separated each condition and was jittered to last on average 6 s. Again, a 12 s (4 TR) dummy run was included at the beginning of each functional run to allow for the scanner magnetization to reach a steady state. Participants completed the same attentional task as the pRF runs throughout the experiment.

TCS data were analysed using MATLAB 2016a and VISTA software. A general linear model (GLM) was implemented to test the contribution of stimulus condition to the BOLD time course (Friston et al., 1998). We used the default two-gamma Boynton HRF from SPM5 and fit the model to an averaged time course of BOLD signal changed for each stimulus condition by minimizing the sum of squared errors (RSS) between the predicted time series and the measured BOLD response. This resulted in 20 Beta weight estimates for each voxel, reflecting sensitivity to each stimulus condition.

### 2.5 Statistical analysis

#### 2.5.1 Plotting beta weights as a function of eccentricity and pRF size

Only pRF and TCS voxels with ≥10% variance explained were retained for further analysis. The pooled total voxel count for each ROI and the total voxels removed for falling below 10% variance explained are presented in Table 1. For each voxel within each participant's ROI, a pRF eccentricity value and a pRF size value was extracted from the pRF data. The same ROIs were then overlaid on each corresponding participants TCS data and 20 beta weights (1 beta weight per stimulus condition) were extracted for each voxel. Thus, each voxel was allocated 22 values: a pRF eccentricity value, a pRF size value, and 20 beta weights reflecting voxel sensitivity to each TCS stimulus condition. Polar angle values were not included in the analysis.

**Table 1:** Results of voxel thresholding. Voxels with less than 10% VE in both the pRF and the TCS data are removed from further analysis (N = 19).

ROI	Pooled total voxels	Pooled voxels under 10% VE	Proportion removed
V1	77693	34314	44.16%
V2	76991	32555	42.28%
V3	70977	26907	37.81%
V3a	55659	23235	41.75%
hV4	25388	25388	49.59%

**Fig. 5:**
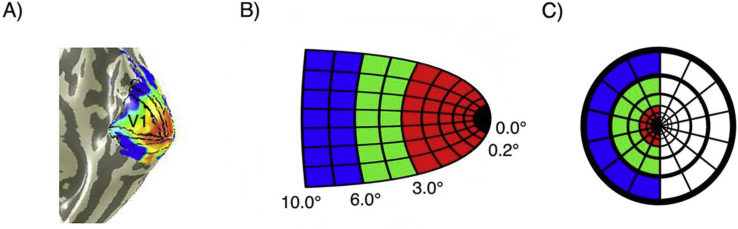
Voxels are binned into 3 gradients of eccentricity – foveal (red), parafoveal (green), and peripheral (blue). In A) we present an eccentricity map on a right hemisphere mesh of the visual cortex with overlaid hand drawn ROIs, noting the location of V1. B) shows how these voxel bins would be represented on a schematic model of right hemisphere V1. In C) we present how the voxel bins in B) would be spatially tuned (ignoring polar angle) across the contralateral visual field.

For each participant, beta weights were plotted as a function of pRF eccentricity; foveal, parafoveal, or peripheral. For each ROI, foveal pRFs were defined as being between 0.2° and 3.0° eccentricity, parafoveal pRFs were defined as being between 3.0° and 6.0° eccentricity, and peripheral pRFs were defined as being between 6.0° and 10.0° eccentricity. Visualisation of how these data are partitioned and their correspondence to visual space is illustrated in Fig. 5.

pRF size and eccentricity are highly related measures: average pRF sizes increase with eccentricity (Dumoulin and Wandell, 2008). For completeness, we additionally analysed our data as a function of pRF size to complement the eccentricity-based analysis. Each participant's beta weights were plotted as a function of pRF size; small or large. Receptive field sizes progressively increase as one moves up the visual hierarchy and what constitutes a ‘small’ or ‘large’ pRF will differ depending on ROI (Wandell et al., 2007). To account for this, within each ROI, ‘small pRFs’ were defined as having a size value between 0.25° (as a hard minimum) and the median pRF size, whilst ‘large pRFs’ were defined as a size value between the median and the maximum pRF size (with a maximum cut off of 10°). These normalized pRF sizes are presented in Appendix Table A1 and the pRF size analysis is presented in the Supplementary Materials.

### 2.6 Contrast response functions

For each participant's ROIs, hyperbolic ratio functions were fitted at each of the four temporal frequencies for each eccentricity partition of data. We modelled contrast response using the following equation:

$$R\left(\text{C}\right)=\;R_{0}+R_{\text{max}}\;\frac{c^{n}}{c_{50}^{n}+\;c^{n}}$$

Where C is stimulus contrast, $R_{0}$ is the baseline response, $R_{\text{max}}$ is the maximum response rate, $c_{50}$ is the semi saturation contrast, and the exponent, *n*, is the rate at which changes occur and was held at 2 (Albrecht and Hamilton, 1982; Boynton et al., 1999). This resulted in four contrast response functions (CRFs) per ROI at each eccentricity for each participant (i.e. each participant had four CRFs within V1 foveal, four CRFs within V1 parafoveal, and four CRFs within V1 peripheral).

**Fig. 6:**
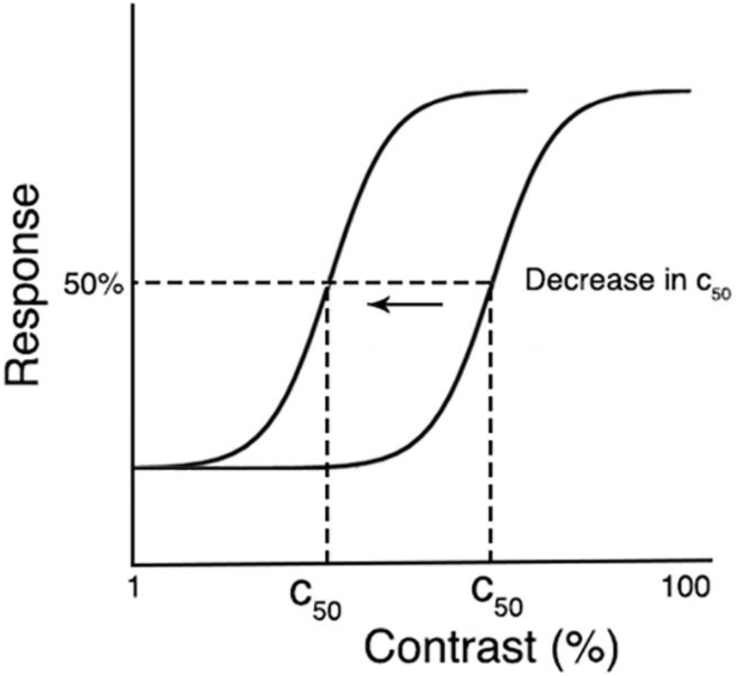
C_50_ plotted on two contrast response functions. C_50_ decreases when the CRF is shifted left, thus less contrast is needed to hit 50% of the full response, reflecting an *increase* contrast sensitivity.

From each CRF we extracted C_50_, the contrast semisaturation point. This is the amount of contrast required to elicit half the maximum response of the CRF. A decrease in C_50_ results in a leftward shift in the CRF, indicating that *less* contrast is required to hit this 50% response, thus, is representative of an *increase* in contrast sensitivity (Albrecht and Hamilton, 1982). Illustration of such a shift in C_50_ is presented in Fig. 6.

### 2.7 Analysis - repeated measures ANOVAs

For our psychophysical experiment, we carried out a 4 (temporal frequency) x 2 (eccentricity) repeated measures ANOVA with 75% contrast detection thresholds as the dependent variable and looked at simple effects to compare between conditions. For our fMRI experiment we ran a 5 (ROI) x 4 (temporal frequency) x 3 (pRF eccentricity) repeated measures ANOVA with C_50_ as the dependent variable and looked at simple effects analyses to answer our targeted predictions.

### 2.8 Polynomial fits and bootstrapping

To find the temporal frequency at which contrast sensitivity peaks at each eccentricity and within each ROI (or for psychophysics, at the two visual field locations tested), we used MATLAB function ‘bootstrp’ to bootstrap 2000 second order polynomial fits (generated using MATLAB function ‘polyfit’) to the means of random permutations of our C_50_ data (fMRI) and contrast detection thresholds (psychophysics). These data were permutated using random sampling (19 draws) with replacement. We then found the mean of the zero points of the first derivatives of each of the 2000 second order polynomial fits. This point reflects the average level of temporal frequency at which contrast sensitivity peaks.

## 3 Results

Our psychophysical data were broadly consistent with those from previous studies indicating little difference in temporal frequency tuning between fovea and near-periphery, and an overall ‘U’ shaped temporal frequency threshold tuning function with a minimum contrast threshold (peak sensitivity) around 8 Hz. In our imaging data, we found profound changes in C_50_ as a function of both temporal frequency and pRF eccentricity. First, we found all visual areas studied had an overall (i.e. ignoring any effects of eccentricity) peak in contrast sensitivity at 10 Hz. Next, in early visual areas we found that pRFs representing the peripheral visual field had increased contrast sensitivity at a high temporal frequency (20 Hz) when compared to pRFs representing the fovea – consistent with effects predicted from retinal physiology. This difference disappeared in area hV4, where no consistent eccentricity-dependent difference in contrast sensitivity at any temporal frequency could be measured. We fed our 20 Hz C_50_ measurements from all ROIs into a linear model and found that hV4 had the highest contribution to a fit of psychophysical contrast sensitivity. Overall, we find that contrast sensitivity in the periphery of V1, V2, V3, and V3a is increased at a high temporal frequency, but this sensitivity is lost in hV4 as cortical tuning becomes more similar that of the psychophysical observer. Here we present a summary of our results for our psychophysical and fMRI data. Supporting pRF size results are available in Supplementary Materials.

### 3.1 Psychophysical results: contrast sensitivity

A 2 x 4 repeated measures ANOVA was performed to assess whether there was a difference in psychophysical contrast detection thresholds between eccentricity and temporal frequency. Mauchly's test of Sphericity was violated for both the main effect of temporal frequency (χ2(5) = 42.321, *p* < .001) and the temporal frequency * eccentricity interaction effect (χ2(5) = 11.619, *p* = .041). Thus, a Greenhouse-Geisser correction was applied to the results of these effects.

**Fig. 7:**
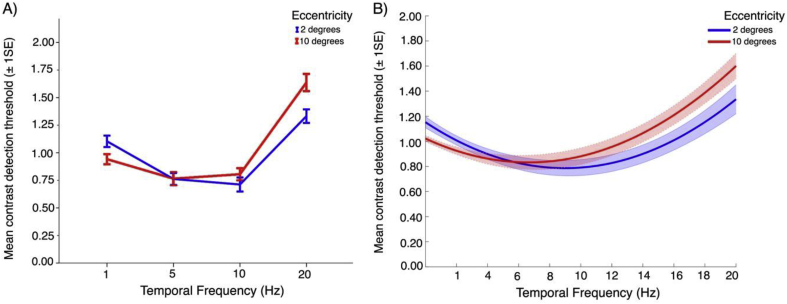
Psychophysical contrast detection thresholds plotted as a function of temporal frequency, at two eccentricities. In A) we present contrast detection thresholds plotted at four measured temporal frequencies at 2° and 10°. In B) we present bootstrapped fits to contrast detection thresholds plotted as a function of temporal frequency at 2° and 10°. Overall, there is little difference in sensitivity at each temporal frequency between fovea and near periphery.

The analysis found a significant main effect of temporal frequency (*p* < .001) and a significant eccentricity * temporal frequency interaction effect (*p* < .001). F-values and *p*-values are presented in Appendix Table A.2. As illustrated in Fig. 7A, contrast detection thresholds were higher at 1 Hz when presented at 2° eccentricity (*p* < .000). Conversely, at 20 Hz, contrast detection thresholds were higher at 10° eccentricity (*p* < .000). Thresholds significantly differed as a function of temporal frequency across both eccentricities, except for comparing between 5 Hz and 10 Hz. All *p*-values are presented in Appendix Tables A.3 and A.4.

### 3.2 Psychophysical temporal frequency optima

To find the temporal frequency at which contrast sensitivity peaks, we looked at the mean zero point of the first derivatives of bootstrapped polynomial fits to our psychological threshold data. At 2° eccentricity contrast sensitivity peaked at 9 Hz, while at 10° eccentricity contrast sensitivity peaked at 6.6 Hz. Bootstrapped fits are presented in Fig. 7B and mean zero points are presented in Appendix Table B1.

### 3.3 fMRI results

A 5 x 4 x 3 repeated measures ANOVA was performed to assess whether there was a difference in contrast sensitivity between ROIs, temporal frequency, and eccentricity. Mauchly's test of Sphericity was violated for the main effect of ROI (χ2(5) = 22.062, *p* = .009) and the interaction effects for ROI * eccentricity (χ2(35) = 52.540, *p* = .036), ROI * temporal frequency (χ2(77) = 121.003, *p* = .003), and eccentricity * temporal frequency (χ2(20) = 42.136, *p* = .003). Thus, a Greenhouse-Geisser correction was applied to the results of these effects. The analysis found significant main effects for eccentricity (*p* = .004) and temporal frequency (*p* = .007). F-values, *p*-values, and effect sizes for main and interaction effects are presented in Appendix Table A.5.

### 3.4 Contrast sensitivity peaks around 10 Hz in all ROIs

**Fig. 8:**
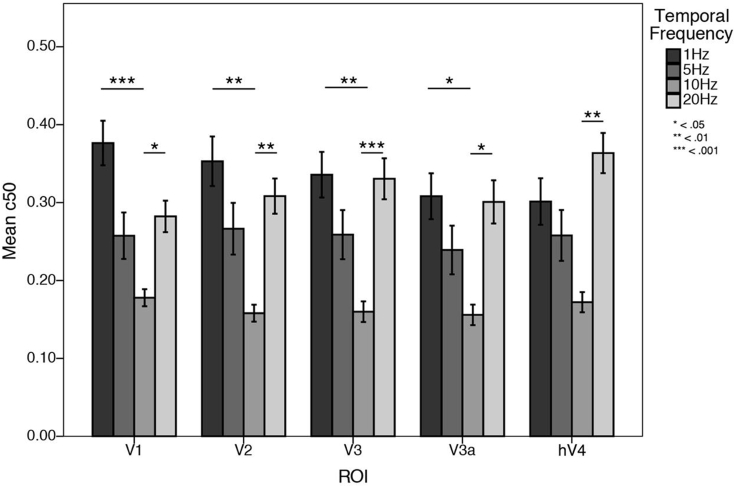
Mean C_50_ values plotted as a function of temporal frequency for each ROI. C_50_ is consistently reduced at 10 Hz in all ROIs, indicating contrast sensitivity peaks at 10 Hz in all visual areas tested.

First, we used a simple effects analysis to explore differences in contrast sensitivity by comparing between the four temporal frequencies, collapsed across pRF eccentricity, within each individual ROI. Sidak corrections were applied to all possible comparisons. As presented in Fig. 8, V1, V2, V3, and V3a had significantly reduced C_50_ at 10 Hz when compared to 1 Hz and 20 Hz (*p* < .05), reflecting increased contrast sensitivity at this temporal frequency. In hV4, C_50_ was significantly reduced at 10 Hz when compared to 20 Hz (*p* = .004). *P*-values for these simple effects are presented in Appendix Table A.6.

### 3.5 fMRI temporal frequency optima

**Fig. 9:**
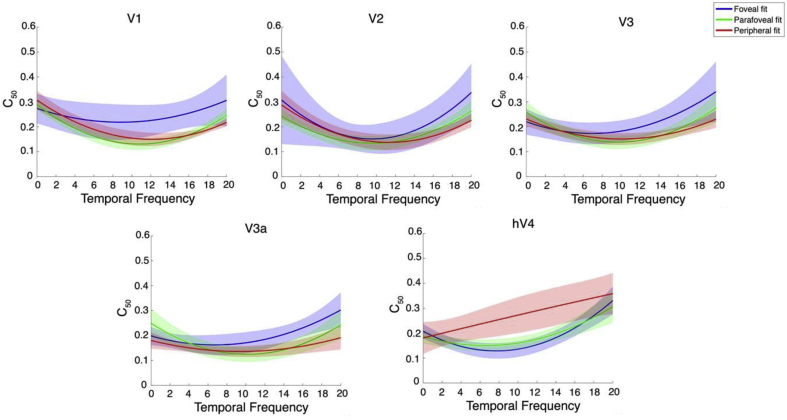
Examples of bootstrapped polynomial fits to C_50_ values plotted as a function of temporal frequency for each eccentricity in all ROIs. The solid line is a second-order bootstrapped polynomial fit to the data and the shaded outline is the standard deviation of 2000 permutations.

**Fig. 10:**
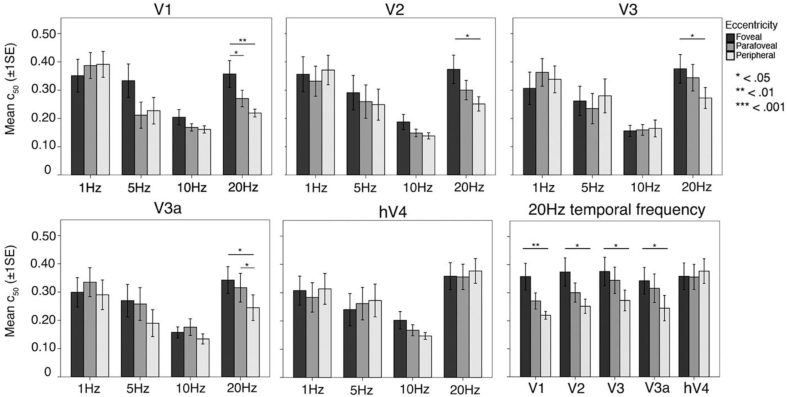
Mean C_50_ values plotted as a function pRF eccentricity at each temporal frequency, for each ROI. In V1—V3a, C_50_ is significantly reduced at 20 Hz in peripheral pRFs, reflecting increased contrast sensitivity at 20 Hz in the cortical periphery. This effect disappears in hV4, where C_50_ is flat across eccentricity at each temporal frequency.

As we did with our psychophysical data, we looked at the mean zero point of the first derivatives of the bootstrapped polynomial fits to our C_50_ values to find, for each ROI and eccentricity, the temporal frequency at which contrast sensitivity peaks. These zero points are presented in Appendix Table B.2 and examples of bootstrapped fits are illustrated in Fig. 9. In V1 and V2, the optimal temporal frequency gradually increased with eccentricity. However, in V3 and V3a the optimal temporal frequency increased from foveal to parafoveal. In hV4 the optimal temporal frequency is essentially identical between the foveal and parafovea. Fits to the data in the periphery of hV4 (see hV4 of Fig. 9) were almost linear and no peak could be computed reliably. We attribute this to variability within the hV4 C_50_ estimates that were derived from the bootstrapping procedure. Thus, the peripheral hV4 fits presented here appear to differ when compared to the corresponding mean hV4 C_50_ values as presented in Fig. 10.

### 3.6 Peripherally tuned pRFs have increased contrast sensitivity at 20 Hz in V1, V2, V3, and V3a

**Fig. 11:**
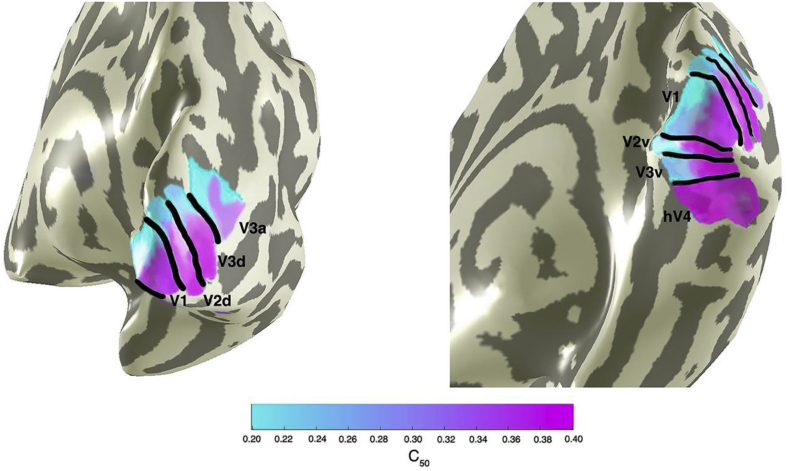
Mean contrast sensitivity maps at 20 Hz projected onto a cortical mesh (N = 19). Early visual field maps V1—V3a show decreasing C_50_ (indicating increasing contrast sensitivity) with increasing eccentricity, whilst contrast sensitivity in hV4 is invariant (and relatively low) across space.

A simple effects analysis was undertaken to explore differences in contrast sensitivity within each ROI at each temporal frequency, comparing between foveal, parafoveal, and peripherally tuned pRFs. Sidak corrections were applied to all possible comparisons. Mean C_50_ values at all temporal frequencies and at 20 Hz alone are presented in Fig. 10. We found eccentricity-dependent differences in contrast sensitivity at 20 Hz. Namely, we found that in V1, V2, V3, and V3a, C_50_ at 20 Hz was consistently decreased in peripherally tuned pRFs when compared to foveally tuned pRFs (*p* < .05), reflecting increased contrast sensitivity at a high temporal frequency in the cortical periphery. There was no difference in contrast sensitivity as a function of eccentricity at 1, 5, or 10 Hz, in any ROI. In Fig. 11 we present a surface-based average (N = 19) contrast sensitivity map at 20 Hz, projected onto an inflated cortical mesh. Similar to previous psychophysical sensitivities, contrast sensitivity in hV4 was invariant across eccentricity at all temporal frequencies tested, including 20 Hz. All *p*-values are presented in Appendix Table A.7.

### 3.7 Comparing psychophysical and fMRI contrast sensitivities

**Fig. 12:**
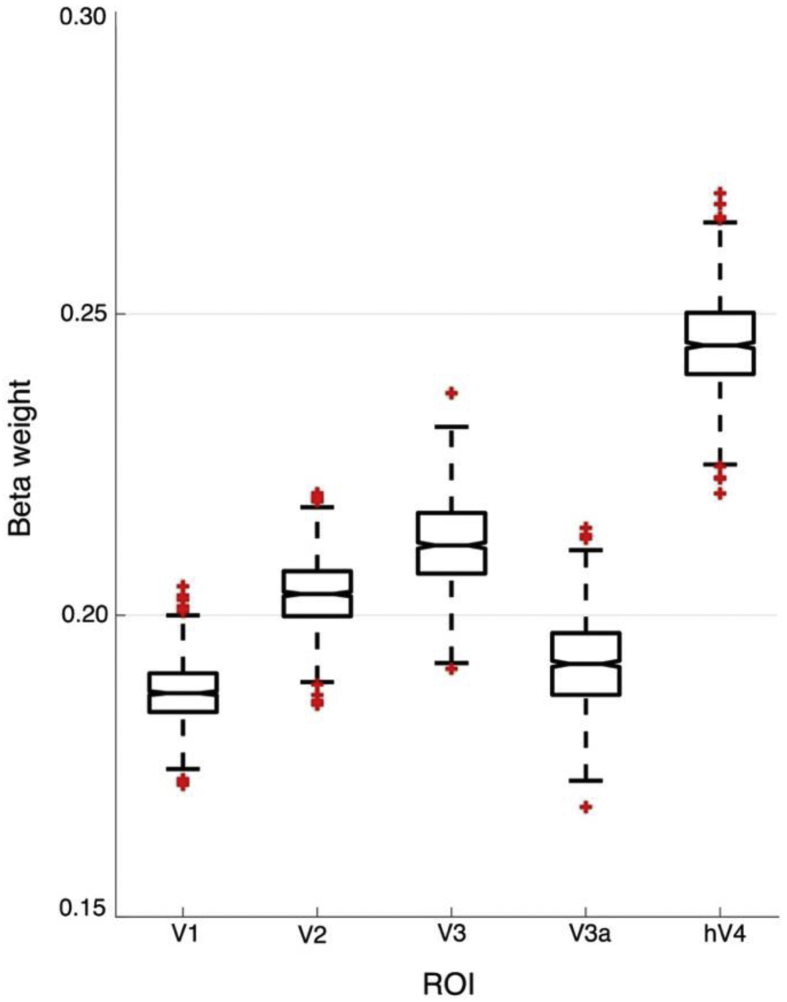
Median bootstrapped beta weights after predicting a fit of psychophysical contrast sensitivity using C_50_ measurements at 20Hz from each ROI. hV4 has the highest beta weight, indicating that this region is the best predictor of psychophysical contrast sensitivity at 20 Hz.

Unlike earlier visual areas, we found that contrast sensitivity at 20 Hz in hV4 was relatively invariant across eccentricity. This finding is more similar to psychophysical sensitivities from our own and other behavioural studies that report little difference in temporal contrast sensitivity across visual space (Koenderink et al., 1978; Virsu et al., 1982; Wright and Johnston, 1983). Next, we aimed to examine the relationship between psychophysical performance and fMRI signals driven by 20 Hz stimuli. Here, we bootstrapped 1000 estimates of 20 Hz fMRI C_50_ measurements from the fovea and periphery of each ROI, and fed this data into a linear model to assess how each ROI contributed to a fit of psychophysical contrast sensitivity at 20 Hz. As illustrated in Fig. 12, we found that C_50_ values from hV4 contributed proportionally more to our psychophysical measurements when compared to early visual areas, indicating that fMRI responses from this area best predict our psychophysical measurements. Bootstrapped beta weight statistics are available in Appendix Table B.3.

## 4 Discussion

We have measured differences in psychophysical and cortical contrast sensitivity that occur as a function of temporal frequency and visual field eccentricity. Overall, our findings indicate that both psychophysical and cortical contrast sensitivity follow a ‘U’ shape function and is maximal between 8 and 12 Hz across visual space. Further, in early visual areas there is a relative increase in contrast sensitivity at 20 Hz in pRFs tuned to more peripheral regions of the visual field. We discuss these findings in light of the physiological bias towards faster visual processing and increased contrast sensitivity in the peripheral retina. As we progressed up the visual pathway to visual area hV4, we observed an equalisation of temporal contrast sensitivity across eccentricity that was closer to psychophysical measurements, suggesting that the peripheral bias in retinal temporal contrast sensitivity disappears in this cortical area.

### 4.1 Peak psychophysical and fMRI contrast sensitivity

Previous research has typically measured the primate visual system's sensitivity to temporal frequency at a single level of contrast. These studies invariably identify a bandpass peak in temporal sensitivity occurring at approximately 8 Hz (Hawken et al., 1996; Kastner et al., 2004; Kwong et al., 1992; Robson, 1966; Singh et al., 2000; Venkataraman et al., 2017). Our approach was similar to these studies, except that we fit a CRF to a range of contrasts presented at different temporal frequencies, then defined our measurement of contrast sensitivity as 50% of the full CRF response (C_50_). Our data showed a similar bandpass pattern. Peak psychophysical contrast sensitivity occurred at 9 Hz and 6.6 Hz at 2° and 10° eccentricity, respectively. Similarly, in our fMRI data we found contrast sensitivity generally peaked around 8 Hz, with the critical frequency of this peak increasing between foveal and peripheral voxels. In this respect, the overall ‘U’ shape of our behavioural and cortical contrast sensitivity functions appears to be matched from a relatively early stage in the visual hierarchy.

Perhaps surprisingly, previous research has found little change in psychophysical temporal contrast sensitivity as a function of eccentricity (Koenderink et al., 1978; Rovamo and Raninen, 1984; Virsu et al., 1982). Although our own psychophysical data showed a slight decrease in temporal contrast sensitivity from the fovea to the near periphery, these differences were relatively small and may reflect difficulties in compensating precisely for cortical magnification effects or stimulus sizing in our own psychophysics (Granit and Harper, 1930; Hassan et al., 2016).

### 4.2 Peripherally tuned pRFs have increased contrast sensitivity at 20 Hz

Physiological biases in the response properties of retinal cells lead to increased temporal contrast sensitivity in more peripheral regions of the retina. Peripheral cones respond faster than foveal cones, resulting in greater peripheral sensitivity to rapidly changing input (Sinha et al., 2017). There is also an eccentricity-dependent increase in the ratio of parasol to midget RGCs, and parasol cells are relatively more sensitive to high temporal frequencies and have increased contrast gain when compared to midget cells (Connolly and van Essen, 1984; Dacey, 1993, 1994; Dacey and Petersen, 1992; De Monasterio and Gouras, 1975; Schein and de Monasterio, 1987). At 10° eccentricity, measurements of temporal contrast sensitivity are thought to reflect more isolated functions of parasol RGCs (Croner and Kaplan, 1995; Gouras, 1968; Kaplan et al., 1990; Kaplan and Shapley, 1986). Signals passed from RGCs pass through the LGN, where the density of afferent parasol and midget RGCs is maintained, before being sent to primary visual cortex (Connolly and van Essen, 1984; Schein and de Monasterio, 1987). Our data show that a sensitivity bias similar to that found in the retina and LGN is present in early visual cortex, with relatively increased contrast sensitivity at 20 Hz in peripherally tuned voxels.

It is well known that neuronal spatial frequency sensitivity tends to be inversely related to temporal frequency sensitivity, thus, channels sensitive to low spatial frequencies are often sensitive to higher temporal frequencies (and vice versa). In addition, the sensitivity of these channels changes as a function of eccentricity (D'Souza et al., 2016; Henriksson et al., 2008; Kulikowski and Tolhurst, 1973; Shoham et al., 1997; Sun et al., 2007). Here, we report measurements made at a single spatial frequency (1 cpd). This frequency was chosen because it is well below the spatial resolution limit at the highest eccentricities measured, yet generates robust responses in the fovea (D'Souza et al., 2016; Henriksson et al., 2008; Welbourne et al., 2018). It is possible that our results would change if a different spatial frequency was used: altering the base spatial frequency might, for example, alter the balance of parvo-to magnocellular cells contributing to the stimulus at each eccentricity, which would, in turn, alter the average temporal response properties (Levitt et al., 2001).

### 4.3 hV4 is similar to the psychophysical observer

Unlike earlier visual areas, we found that temporal contrast sensitivity does not significantly differ as a function of eccentricity in hV4. Specifically, there appears to be little bias towards higher temporal contrast sensitivity in more peripheral regions of hV4. Instead, temporal contrast sensitivity in hV4 is more reflective of the behavioural observer. After bootstrapping a linear model to assess the contribution of our 20 Hz C_50_ data to a fit of psychophysical measurements, we found that hV4 had a propotionally greater contribution to psychophysical sensitivities when compared to all other visual areas. It may be that higher order areas become increasingly invariant to eccentricity-dependent differences in low-level features, including contrast and temporal frequency, and instead represent more complex stimulus aspects relating to shape, identity, and colour (Avidan et al., 2002; Felleman and Van Essen, 1991; Milner and Goodale, 1995; Perry and Fallah, 2014). For example, hV4 has previously been found to have a much coarser representation of spatial frequency and an increased tolerance to temporal dynamics when compared to earlier visual areas, suggesting these areas are less concerned with such low level visual properties (Henriksson et al., 2008; Zhou et al., 2017). In a similar vein, ventral regions local to hV4 that are concerned with global form and object representations such as FFA, PPA, VO, and LOC, have at times found to be invariant to lower level visual features, and fMRI responses within such regions can become impaired when stimuli are presented at high temporal frequencies (D'Souza et al., 2011; Grill-Spector et al., 1999; Jiang et al., 2007; Kanwisher, 2010; Liu and Wandell, 2005; Mckeeff et al., 2007; Vernon et al., 2016). Although this bias in retinal temporal contrast sensitivity is phased out by hV4, our data found that this area also responds optimally around 10 Hz temporal frequency – perhaps inheriting this sensitivity bias from earlier regions.

## 5 Conclusion

Our experiments have found that in general, psychophysical and fMRI measurements of contrast sensitivity are relatively consistent and both peak around 8 Hz. Next, pRFs in early visual areas that represent more peripheral regions of visual space show relatively increased contrast sensitivity at a high temporal frequency when compared to those in the cortical representation of the fovea. However, this bias in peripheral cortical contrast sensitivity disappears by hV4, suggesting a relative independence of temporal contrast sensitivity across space in this area. This independence is broadly consistent with behavioural measurements of temporal contrast sensitivity, and suggests that neurons in area hV4 (and possibly other higher-order ventral regions) are relatively invariant to the eccentricity-dependent biases that are present in the early visual stream.

## Author contributions

M.M.H and A.R.W conceived and designed the experiments, M.M.H performed the experiments, M.M.H and A.R.W analysed the data, M.M.H wrote the paper, and M.M.H and A.R.W revised the paper.

## Declaration of interest

The authors declare no competing financial interests.

## Figures and Tables

**Table A.1:** Normalized pRF sizes for each ROI (N = 19). For each ROI, small pRFs fall between the minimum and median pRF size, and large pRFs fall between median and maximum pRF size.

Visual Area	Min pRF size	Median pRF size	Max pRF size
V1	0.25°	1.72°	9.89°
V2	0.25°	2.06°	9.30°
V3	0.25°	2.89°	9.74°
V3a	0.25°	4.06°	10.0°
hV4	0.25°	4.7°	10.0°

**Table A.2:** Tests of within-subjects effects for psychophysical data. Temporal frequency and eccentricity as IVs, and contrast detection threshold as DV.

Source	*df*	F	pη^2^	*p*
Temporal Frequency (GG)	1.895	88.179	.830	.000***
Eccentricity	1	3.824	.175	.066
TF*Eccentricity (GG)	2.210	23.459	.566	.000***

**Table A.3:** Simple effects comparisons for psychophysical data. Differences in contrast detection thresholds, comparing between two factors of eccentricity at each temporal frequency (N = 19).

Temporal Frequency		10°
1 Hz	2°	.000***
5 Hz	2°	.946
10 Hz	2°	.057
20 Hz	2°	.000***

**Table A.4:** Simple effects comparisons for psychophysical data. Differences in contrast detection thresholds, comparing between four factors of temporal frequency at each eccentricity (N = 19).

Eccentricity		5 Hz	10 Hz	20 Hz
2°	1 Hz	.000***	.000***	.023*
	5 Hz	–	.324	.000
	10 Hz	–	–	.000***
10°	1 Hz	.000***	.019*	.000***
	5 Hz	–	.277	.000***
	10 Hz	–	–	.000**

**Table A.5:** Tests of within-subjects effects for fMRI data. ROI, eccentricity, and temporal frequency as IVs, and C_50_ as DV (N = 19).

Source	*df*	*F*	*p*	*power*
ROI (GG)	2.749	.684	.554	.177
Eccentricity	2	6.403	.004**	.875
TF	3	4.466	.007**	.853
ROI*Eccentricity (GG)	4.334	2.158	.077	.838
ROI*TF (GG)	5.977	1.638	.145	.602
Eccentricity*TF (GG)	2.911	2.132	.110	.504
ROI*Eccentricity*TF	24	1.314	.148	.927

**Table A.6:** Simple effects for fMRI data. Differences in C_50_, comparing between four factors of temporal frequency within each ROI (N = 19).

Visual Area		5 Hz	10 Hz	20 Hz
V1	1 Hz	.281	.000***	.295
	5 Hz	–	.287	.999
	10 Hz	–	–	.010*
V2	1 Hz	.682	.006**	.960
	5 Hz	–	.279	.990
	10 Hz	–	–	.004**
V3	1 Hz	.676	.007**	1.000
	5 Hz	–	.449	.909
	10 Hz	–	–	.007*
V3a	1 Hz	.813	.045*	1.000
	5 Hz	–	.642	.919
	10 Hz	–	–	.037*
hV4	1 Hz	.969	.124	.924
	5 Hz	–	.595	.549
	10 Hz	–	–	.004**

**Table A.7:** Simple effects for fMRI data. Differences in C_50_, comparing between three factors of eccentricity at each temporal frequency within each ROI (N = 19).

			Parafoveal	Peripheral
V1	1 Hz	Foveal	.913	.900
		Parafoveal	–	.994
	5 Hz	Foveal	.072	.072
		Parafoveal	–	.963
	10 Hz	Foveal	.284	.136
		Parafoveal	–	.358
	20 Hz	Foveal	.026*	.008**
		Parafoveal	–	.061
V2	1 Hz	Foveal	.993	.995
		Parafoveal	–	.763
	5 Hz	Foveal	.827	.585
		Parafoveal	–	.763
	10 Hz	Foveal	.302	.222
		Parafoveal	–	.214
	20 Hz	Foveal	.319	.046*
		Parafoveal	–	.067
V3	1 Hz	Foveal	.566	.922
		Parafoveal	–	.864
	5 Hz	Foveal	.755	.893
		Parafoveal	–	.393
	10 Hz	Foveal	.999	.996
		Parafoveal	–	.997
	20 Hz	Foveal	.592	.034*
		Parafoveal	–	.086
V3a	1 Hz	Foveal	.512	.938
		Parafoveal	–	.186
	5 Hz	Foveal	.843	.191
		Parafoveal	–	.272
	10 Hz	Foveal	.889	.610
		Parafoveal	–	.420
	20 Hz	Foveal	.395	.016*
		Parafoveal	–	.033*
hV4	1 Hz	Foveal	.895	.997
		Parafoveal	–	.431
	5 Hz	Foveal	.957	.953
		Parafoveal	–	.995
	10 Hz	Foveal	.481	.118
		Parafoveal	–	.174
	20 Hz	Foveal	1.000	.928
		Parafoveal	–	.942

**Table B.1:** Bootstrapped descriptive statistics psychophysical data (2000 iterations). Mean of the zero points of the first derivative of our bootstrapped fits to contrast threshold data, which is representative of the temporal frequency at which psychophysical contrast sensitivity peaks.

	Bootstrap Distribution Mean	Bootstrap Distribution Median	SD
2°	9.00	8.92	.58
10°	6.60	6.54	.57

**Table B.2:** Bootstrapped descriptive statistics for fMRI data (2000 iterations). Mean of the zero points of the first derivative of our bootstrapped fits to C_50_ data, which is representative of the temporal frequency at which fMRI contrast sensitivity peaks.

		Bootstrap Distribution Mean	Bootstrap Distribution Median
V1	Foveal	8.41	8.24
	Parafoveal	10.60	10.55
	Peripheral	12.10	12.03
V2	Foveal	7.59	7.34
	Parafoveal	9.10	9.06
	Peripheral	11.13	11.33
V3	Foveal	6.17	6.99
	Parafoveal	9.63	9.31
	Peripheral	9.53	9.47
V3a	Foveal	5.88	6.25
	Parafoveal	9.55	9.42
	Peripheral	9.16	9.02
hV4	Foveal	7.01	7.01
	Parafoveal	5.30	5.75
	Peripheral	–	–

**Table B.3:** Bootstrapped beta weight estimates (1000 iterations) after feeding foveal and peripheral 20Hz C_50_ values into a linear model to assess how each ROI contributed to a fit of psychophysical contrast sensitivity.

ROI	Bootstrap Median Beta Weight
V1	0.18
V2	0.21
V3	0.22
V3a	0.19
hV4	0.24

## Appendix C Supplementary data

SupplementaryMaterials_Final SupplementaryMaterials_Final
