# Supplementary material for: Eccentricity-dependent temporal contrast tuning in human visual cortex measured with fMRI
Source: Neuroimage. 2019 Jan 1;184:462–74. doi: 10.1016/j.neuroimage.2018.09.049 (PMC6264386; doi:10.1016/j.neuroimage.2018.09.049)
Supplement: SupplementaryMaterials_Final [file mmc1.docx]

**Supplementary Material:** Temporal contrast tuning as a function of pRF size

Both fMRI and electrophysiological research indicates that receptive field sizes generally become larger with increasing eccentricity (Wandell et al., 2007; Wandell & Winawer, 2015). In our own data we found that pRF size was positively correlated with visual field eccentricity in all ROIs (see Figure 13). In addition to an effect of pRF eccentricity, we asked whether similar biases in temporal contrast sensitivity would occur when we partitioned our data into pRF size. To investigate this, we partitioned our data into small and large pRF sizes to produce similar, although not identical, subsets of voxels and found similar effects when compared to pRF eccentricity partitioned data.


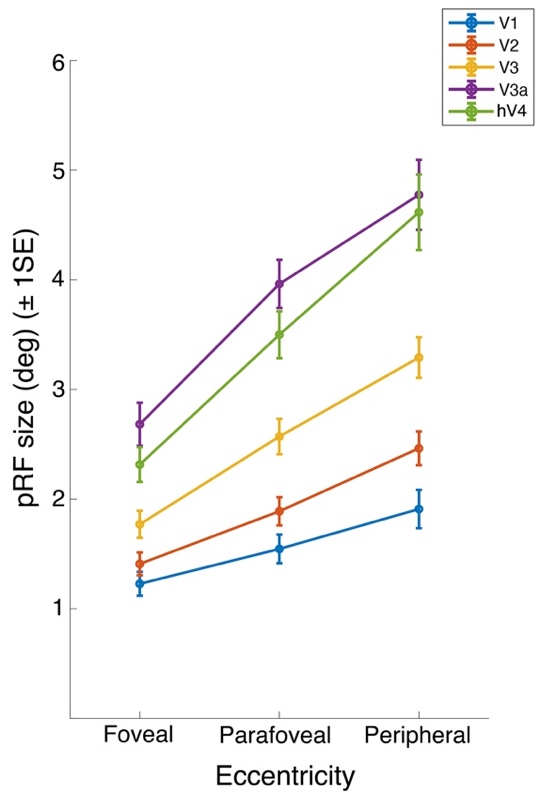


**Figure 13**: Average pRF sizes plotted against three levels of eccentricity. pRF sizes become larger as one moves towards the periphery.

#### Results

A 5 x 4 x 2 repeated measures ANOVA was performed to assess whether there was a difference in contrast sensitivity between ROIs, temporal frequency, and pRF size. Mauchly’s test of Sphericity was violated for the main effect of ROI (χ2(9) = 20.736, *p =* .014) and the interaction effects for pRF size * temporal frequency (χ2(5) = 14.710, *p =* .012) and ROI * temporal frequency * pRF size (χ2(77) = 134.832, *p* < .000). Thus, a Greenhouse-Geisser correction was applied to the results of these effects.

The analysis found significant main effects of pRF size (*p* < .000), temporal frequency (*p* = .003), and a significant interaction effect between ROI * temporal frequency (*p* < .000). F-values, *p*-values, and effect sizes are presented in Table 2.

**Table 2.**

Tests of within-subjects effects for fMRI C_50_ data. ROI, temporal frequency, and pRF size as IVs and C_50_ as DV (N=19).

|  | *df* | *F* | *p* | power |
| --- | --- | --- | --- | --- |
| ROI (GG) | 2.550 | 1.499 | .231 | .341 |
| pRF size | 1 | 21.651 | .000*** | .993 |
| TF | 3 | 5.261 | .003** | .911 |
| ROI*pRF size | 4 | .258 | .904 | .103 |
| ROI*TF | 12 | 3.442 | .000*** | .997 |
| pRF Size*TF (GG) | 2.080 | 3.004 | .060 | .559 |
| ROI*pRF size*TF (GG) | 6.363 | 1468 | .192 | .569 |

A simple effects analysis was undertaken to explore differences in contrast sensitivity within each ROI and at each temporal frequency, comparing between small and large pRF sizes. Sidak corrections were applied to all comparisons. Mean C_50_ values at all temporal frequencies and at 20Hz alone for small and large pRF sizes within each ROI are presented in Figure 14. Within all ROIs, C_50_ was significantly decreased in large pRFs compared to small pRFs at 20Hz (*p* < .05). Additionally, in V1 we found that C_50_ was decreased large pRFs at 10Hz (*p* = .010) and in V3a C_50_ was significantly decreased in large pRFs at 5Hz (*p* = .016). Our results here differed in hV4, where C_50_ was now significantly decreased in large pRFs compared to small pRFs at 20Hz (*p* = .016). All *p*-values are presented in Table 3.


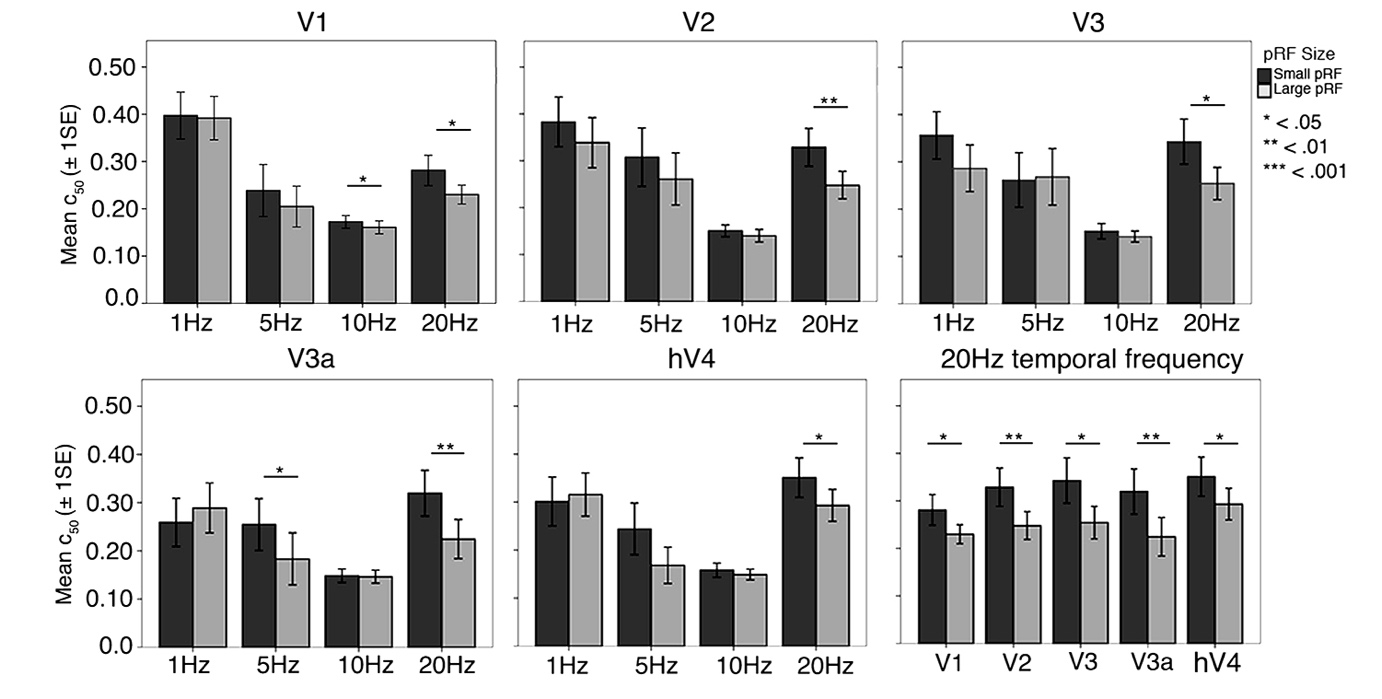


**Figure 14**: Mean C_50_ values plotted as a function of pRF size for each temporal frequency, in each ROI. In all ROIs, C_50_ in significantly reduced at 20Hz in larger pRF sizes.

**Table 3.**

Simple effects for fMRI data. Differences in C_50_ for each ROI, comparing between small and large pRF sizes at each temporal frequency (N=19).

|  |  |  | Large |
| --- | --- | --- | --- |
| V1 | 1Hz | Small | .729 |
|  | 5Hz | Small | .416 |
|  | 10Hz | Small | .010* |
|  | 20Hz | Small | .028* |
| V2 | 1Hz | Small | .336 |
|  | 5Hz | Small | .263 |
|  | 10Hz | Small | .139 |
|  | 20Hz | Small | .005** |
| V3 | 1Hz | Small | .200 |
|  | 5Hz | Small | .735 |
|  | 10Hz | Small | .198 |
|  | 20Hz | Small | .013* |
| V3a | 1Hz | Small | .336 |
|  | 5Hz | Small | .016* |
|  | 10Hz | Small | .780 |
|  | 20Hz | Small | .006** |
| hV4 | 1Hz | Small | .727 |
|  | 5Hz | Small | .093 |
|  | 10Hz | Small | .355 |
|  | 20Hz | Small | .016* |
